# Socio-economic and spatial inequalities in animal sources of iron-rich foods consumption among children 6–23 months old in Ethiopia: A decomposition analysis

**DOI:** 10.1371/journal.pgph.0003217

**Published:** 2024-05-16

**Authors:** Daniel G. Belay, Molla M. Wassie, Melaku Birhanu Alemu, Mehari Woldemariam Merid, Richard Norman, Gizachew A. Tessema

**Affiliations:** 1 Department of Epidemiology and Biostatistics, Institute of Public Health, College of Medicine and Health Sciences, University of Gondar, Gondar, Ethiopia; 2 Curtin School of Population Health, Curtin University, Perth, Western Australia, Australia; 3 Flinders University, College of Medicine and Public Health, Flinders Health and Medical Research Institute, Adelaide, South Australia; 4 Department of Health Systems and Policy, Institute of Public Health, College of Medicine and Health Sciences, University of Gondar, Gondar, Ethiopia; 5 School of Public Health, University of Adelaide, Adelaide, South Australia, Australia; Michigan State University, UNITED STATES

## Abstract

Iron deficiency anaemia is the most common type of anaemia in young children which can lead to long-term health consequences such as reduced immunity, impaired cognitive development, and school performance. As children experience rapid growth, they require a greater supply of iron from iron-rich foods to support their development. In addition to the low consumption of iron-rich foods in low- and lower-middle-income countries, there are also regional and socio-economic disparities. This study aimed to assess contributing factors of wealth-related inequality and geographic variations in animal sources of iron-rich food consumption among children aged 6–23 months in Ethiopia. We used data from the Ethiopian Mini Demographic and Health Surveys (EMDHS) 2019, a national survey conducted using stratified sampling techniques. A total of 1,461 children of age 6–23 months were included in the study. Iron-rich animal sources of food consumption were regarded when parents/caregivers reported that a child took at least one of the four food items identified as iron-rich food: 1) eggs, 2) meat (beef, lamb, goat, or chicken), 3) fresh or dried fish or shellfish, and 4) organs meat such as heart or liver. Concentration indices and curves were used to assess wealth-related inequalities. A Wagstaff decomposition analysis was applied to identify the contributing factors for wealth-related inequality of iron-rich animal source foods consumption. We estimated the elasticity of wealth-related inequality for a percentage change in socioeconomic variables. A spatial analysis was then used to map the significant cluster areas of iron-rich animal source food consumption among children in Ethiopia. The proportion of children who were given iron-rich animal-source foods in Ethiopia is 24.2% (95% CI: 22.1%, 26.5%), with figures ranging from 0.3% in Dire Dawa to 37.8% in the Oromia region. Children in poor households disproportionately consume less iron-rich animal-source foods than those in wealthy households, leading to a pro-rich wealth concentration index (C) = 0.25 (95% CI: 0.12, 0.37). The decomposition model explained approximately 70% of the estimated socio-economic inequality. About 21% of the wealth-related inequalities in iron-rich animal source food consumption in children can be explained by having primary or above education status of women. Mother’s antenatal care (ANC) visits (14.6%), living in the large central and metropolitan regions (12%), household wealth index (10%), and being in the older age group (12–23 months) (2.4%) also contribute to the wealth-related inequalities. Regions such as Afar, Eastern parts of Amhara, and Somali were geographic clusters with low iron-rich animal source food consumption. There is a low level of iron-rich animal source food consumption among children, and it is disproportionately concentrated in the rich households (pro-rich distribution) in Ethiopia. Maternal educational status, having ANC visits, children being in the older age group (12–23 months), and living in large central and metropolitan regions were significant contributors to these wealth-related inequalities in iron-rich animal source foods consumption. Certain parts of Ethiopia such as, Afar, Eastern parts of Amhara, and Somali should be considered priority areas for nutritional interventions to increase children’s iron-rich animal source foods consumption.

## Background

The most common dietary deficiencies leading to serious developmental issues worldwide are micronutrient deficiencies [1]. Anaemia is the most common micronutrient deficiency problem that disproportionately affects preschool-aged children globally [2–4]. Nearly two-fifths (39.8%) of under-five children globally, and three out of five children (60.2%) in Africa were anaemic in 2019 [5]. Based on the 2021 World Health Organization (WHO) global Anaemia report, showed more than half (56%) of children aged 6 to 59 months in Ethiopia were anaemic [5]. Iron deficiency is the most common cause of anaemia [3, 6, 7] and affects approximately 32% of sub-Saharan Africa’s under-five children [8]. Moreover, one-quarter (25%) of children under five in southern parts of Ethiopia were also affected by iron deficiency anaemia [9].

Early childhood iron requirements are influenced by the infant’s rate of growth, iron intake, and iron loss [7]. Given infants grow so quickly, they have higher iron demands than other age groups [10]. Iron is highly needed by infants in the first few months after birth to form red blood cells [7]. Furthermore, infants frequently use iron stored during the foetal period, which can lead to low or exhausted stores by the time they reach 4–6 months of age [11]. Additionally, infants who are breastfed could face a higher susceptibility to iron deficiency due to the relatively low iron content in breast milk [12, 13], especially if breast milk replaces solid foods in the diet [12]. In the 2016 Ethiopian national micronutrient survey as measured by ferritin and adjusted for inflammation, 17.8% of children had depleted iron stores (serum ferritin ≤12 μg/L) [14]. Iron deficiency anaemia due to low iron intake would cause reduced immunity, low cognitive development, and retarded growth in preschool children that can continue throughout their lifetime [15].

Global efforts to reduce anaemia are directed towards increasing the intake of iron-rich food sources since anaemia is commonly attributable to iron deficiency [3]. The WHO recommends consuming iron-rich complementary foods daily, including lean red meats, fish and poultry, and iron-fortified cereals to prevent iron deficiency anaemia (IDA) in young children [16, 17].

However, in sub-Saharan Africa, 42.1% of children consume the recommended serving of iron-rich foods [18]. Moreover, less than half of children regularly consumed iron-rich foods in French-speaking African countries [19]. In Ethiopia, a study using the 2016 Ethiopian Demographic and Health Survey (EDHS 2016) showed that only one in five (21.41%) children aged 6–23 months reported consumption of iron-rich animal sources of foods [4].

Studies showed that household wealth status is one of the major determinants of children’s consumption of iron-rich foods [4] in addition to lack of awareness of iron-rich foods, short interpregnancy spacing [20], feeding culture [21], and low maternal educational status [4].

The high bioavailability of micronutrients in animal sourced foods can be explained by the presence of heme protein in meat, poultry, and fish, along with the absence of inhibitors such as phytate commonly found in plant-based diets such as legumes, cereals, nuts and seeds [14, 22]. Moreover, unlike plant sources of iron foods, animal sources of food contain both heme and non-heme iron and have high absorption in the body [23]. Studies have shown that a higher usual intake of animal sources of food in low-income countries is associated with better growth, status of some micronutrients, cognitive performance, motor development, and activity [24]. However, despite animal dietary sources being high in minerals, protein, and fat, their access and affordability are limited in low and middle-income countries such as Ethiopia [4]. There is a paucity of studies investigating the extent and direction of wealth-related inequalities in animal sources of iron-rich food consumption among children aged 6–23 months in Ethiopia.

Therefore, this study aims to quantify the level of wealth-related and geographic inequalities of iron-rich animal source foods consumption among children aged 6–23 months in Ethiopia. Moreover, the overall wealth-related inequalities of iron-rich food consumption have been decomposed by variables and the contribution of each factor to the observed inequality is estimated. This is important to indicate the possibility of reducing inequalities in iron-rich food consumption among children by narrowing the household income gap and enhancing the economic status of the lowest wealth quantile groups.

## Methods

### Study design, setting, and period

This study is a secondary analysis of data obtained from the Ethiopian Mini Demographic and Health Survey 2018 (EMDHS, 2019). The survey was conducted between March and June 2019 [25]. Ethiopia is an East African country with 1.1 million km^2^ coverage and a population of around 115 million in 2021 [26]. Currently, Ethiopia is federally decentralised into eleven regions and two city administrations [27, 28]. Four regions (Sidama, Central Ethiopia, Southern Ethiopia and Southwest Ethiopia) have been recently formed from Southern Nations, Nationalities, and Peoples’ Region (SNNPR) after the survey was conducted [27], and the results were presented as part of the SNNPR region.

Ethiopia’s health care system employs a three-tier system. The first level is a primary health care unit (PHCU) which comprises four health centres (HCs), five health posts within each health centre, and a primary hospital. Each health post is responsible for a population of 3,000–5,000 people. While the secondary level of care consists of general hospitals. The tertiary level of care comprises specialised hospitals and university hospitals [29].

### Populations and sampling method

The source population was all children aged 6–23 months during the survey period. A stratified two-stage cluster sampling method was used for data collection. Each administrative region was stratified by dividing them into urban and rural areas except Addis Ababa. In total, 21 sampling strata have been created. Enumeration Areas (EAs) or clusters were the sampling units for the first stage of sampling. In the 2019 EMDHS, a total of 305 EAs (93 in urban areas and 212 in rural areas) were selected with probability proportional to EA size (based on the 2019 Ethiopia Population and Housing Census (EPHC) frame) [28, 30]. To ensure that survey precision was comparable across regions, the sample allocation was done through an equal allocation where 25 EAs were selected from six regions (Tigray, Afar, Somali, Benishangul Gumuz, Gambelia, and Harari) and two city administrations (Addis Ababa and Dire Dawa). Whereas 35 EAs were selected from each of the three larger regions: Amhara, Oromia, and the SNNPR. In the second stage, a complete household listing was conducted in each of the selected clusters and a fixed number of households were selected by equal probability systematic sampling in the selected cluster. Since the overall probability of selection of each household was not constant, sample weighting was applied during the analysis.

### Sample size

Of the total of 1,523 eligible children aged 6–23 months during the survey period, 20 children were not living with their mothers, and the other 41 children were not indexed births in the past two years and were excluded from further analysis. To provide regional representative sampling, the DHS survey usually implements weighting during the sampling design [31]. That is, for regions with smaller population sizes, DHS oversampled the number of participants required in the survey. Conversely, for regions with larger population sizes, DHS under-sampled the number of study participants. To account for this, the DHS survey estimated and provided an individual weighting variable for analysis [31]. Finally, all the analyses were conducted using the weighted samples of 1,461 children.

### Study variables

The outcome variable of this study was *animal sources of iron-rich food consumption* (defined as a binary variable) among children 6–23 months in Ethiopia. While iron-source foods contain both animals and plants, our analysis focused on animal-source foods owing to the information available in existing data. Consistent with literature [4, 28], animal sources of iron-rich food consumption were considered when a child of age 6–23 months old living with their mother and reported to consume at least one of the four iron-rich food items within the 24 hours preceding the interview: 1) eggs, 2) meat (beef, lamb, goat, or chicken), 3) fresh or dried fish or shellfish, and 4) organs meat such as heart or liver.

Socio-demographic characteristics such as the mother’s age, marital status, maternal education, number of children under five years old in the family (excluding children who came to visit the family), religion, household wealth index, geographic residence (urban/rural), and administrative were also included.

The wealth index was determined using a household’s ownership of specific assets, as recorded in the household questionnaires of demographic and health survey (DHS). It was generated by considering the following proxy variables: a household’s ownership of specific assets for urban and rural results in households separately. While assets for urban households included televisions, electricity, refrigerators, vehicles, housing features, water sources, toilet facilities etc., the assets for rural areas included ownership of agricultural land and size, farm animals by type and number, housing conditions, furniture etc. [28, 30, 32]. Then separate factor scores were produced for households in urban and rural areas using area-specific indicators. Later, separate area-specific factor scores were combined to produce a nationally applicable combined wealth index by adjusting the area-specific scores through regression on the common factor scores. Each household asset was given weight through principal components analysis, and the scores were standardised [32]. Households were then ranked based on their total scores and divided into five quintiles. Wealth quintiles are defined based on the distribution of individuals in the population, rather than specific health or population indicators [28, 30]. For more details, you can see elsewhere [28].

Moreover, based on the development status and the need for governmental support, the 11 regions of Ethiopia were categorised into three groups; ‘three metropolitan (Addis Ababa, Harari, and Dire Dawa), large central (Tigray, Amhara, Oromia, SNNPR), and “communities with predominately pastoralist regions” (Afar, Benishangul-Gumuz, Gambelia, and Somali) [33]. Child-related factors also included the age of the child, sex of the child, and current breastfeeding status.

### Data processing and analysis

The KR dataset was downloaded in STATA format from the DHS publicly available website (https://dhsprogram.com/data/) on January 10, 2023, and then integrated, transformed, and appended to produce appropriate variables for the analysis. STATA 14 software was used to aggregate variables and generate descriptive and statistical analysis. ArcGIS version 10.7 and SaTScan version 9.6 software were used to assess the spatial distribution of iron-rich animal source foods consumption among children under the age of two in Ethiopia.

### Wagstaff decomposition analysis

To examine the socio-economic inequalities of iron-rich animal source foods consumption, the Wagstaff normalised concentration index and graph approach were used [34, 35].

The concentration index quantifies the extent and direction of socio-economic-related inequalities in an outcome variable (iron-rich animal source foods consumption). It ranges from −1 to + 1, the value of a negative sign indicates the concentration of iron-rich animal source foods consumption among the poor (pro-poor distribution), whereas a positive value indicates concentration among the richer groups (pro-rich distribution). Moreover, the concentration index becomes zero when the concentration curve lies on the line of equality which implies that there are no wealth-related inequalities for the health outcome (iron-rich food consumption) [36].

However, since the outcome variable in this study is binary (consume/not consume), the concentration index’s bounds depend on its mean (μ). As a result, the Wagstaff normalisation correction was used to calculate the normalised concentration index by dividing the concentration index (C) by 1 minus the mean (1–μ) [36, 37].


$${\text{C}}_{\text{Normalised}}=\frac{C}{1-\mu }$$

The concentration index value is graphically expressed by a concentration curve. To plot the concentration curve, we used the cumulative percentage of the study population, ranked by wealth status, as the X-axis, and the cumulative percentage of the study population, ranked by consumption of iron-rich foods, as the Y-axis. The concentration curve at a slope of 1 indicates the absence of inequity while, the concentration curve laying above and below the equality line (45 degrees) indicates that iron-rich food consumption among children is disproportionately concentrated between poor and rich, respectively [37, 38]

The overall wealth-related inequality of iron-rich animal source foods consumption is decomposed by variables. The outcome variable used for the decomposition is wealth-related inequalities of iron-rich food consumption. Socio-demographic factors such as maternal age, maternal education, marital status, breastfeeding status, number of children under five years old in the family, child age, religion, ANC uptake, geographic residence (urban/rural), and administrative region were considered as contributing factors for the inequalities. To decompose the overall wealth-related inequalities of iron-rich food consumption by variables and explain the contribution of each factor to the observed inequality, the Wagstaff decomposition analysis was used [39].

Each variable’s concentration index (C) was decomposed based on regression analysis of the relationship between an outcome variable and a set of determinants. The overall concentration index can be decomposed into ‘k’ social determinant contributions, in which each social determinant’s contribution is obtained by multiplying the sensitivity of the outcome related to that determinant and the degree of income-related inequality in that factor [37, 40].

Based on a linear additive regression model, the concentration index for iron-rich food consumption (C) can be expressed as follows.


$$C=\sum k(\beta k\;\bar{x}k/\mu )\;Ck+GC\varepsilon /\mu$$

Where μ is the mean of C, $\bar{x}k$ is the mean of Xu, Ck is the concentration index of xk, and GCε (residual) is the generalised concentration index for the error term (*ε*). The overall concentration index of iron-rich food consumption includes the explained part which is the sum of the contributions of k determinants, and the unexplained part (residual). Based on the Wagstaff normalisation, the normalised decomposition of the concentration index is obtained by dividing the concentration index by 1–μ [36]. Elasticity is the sensitivity of iron-rich animal sources of food consumption inequality for each factor. The concentration index in each variable is the degree and direction of socio-economic-related inequality in iron-rich food consumption corresponding to specific explanatory variables. Absolute contribution is expressed in the same unit as the C whereas relative contribution is the percentage of the C of each covariate to the total observed income-related inequality in iron-rich food consumption [41].

### Spatial analysis of iron-rich animal source foods consumption in Ethiopia

Global Moran’s I statistic spatial autocorrelation measure was used to assess the geographic distribution of iron-rich animal source foods consumption among children in Ethiopia, using the prevalence of outcome at cluster/EA level [42]. Whereas to predict the iron-rich food consumption among children in Ethiopia for unsampled areas based on sampled clusters a spherical semivariogram ordinary kriging type spatial interpolation technique was used. The proportion of children who consume iron-rich food in each cluster was taken as input for spatial prediction.

Using Kuldorff’s SaTScan version 9.6 software, Bernoulli-based model spatial scan statistics were used to pinpoint the locations of statistically significant clusters for iron-rich animal source food consumption among children under the age of two in Ethiopia [43]. Children who received animal-source iron-rich food consumption were taken as cases and those children who did not receive it were taken as non-cases (controls) to fit the Bernoulli model.

### Ethics approval and consent to participate

This study did not require ethical approval as we used a secondary analysis of a publicly available survey from the Demographic and Health Survey (DHS) program. The authors had no access to information that could identify individual participants during or after data collection.

## Results

### Socio demographic characteristics of mothers

In this study, a total of 1,461 children aged 6–23 months, with a median age of 14 and an interquartile range (IQR) of 10 to 18, were included. The median age of mothers of children was 27 with IQR from 23 to 30. 44.5% of women had no formal education. 75.5% of women had at least one ANC follow up during the pregnancy period of this child. Under one-third (28.4%) of the mothers were urban inhabitants (Table 1).

**Table 1:** Socio-demographic characteristics of selected mothers in Ethiopia.

Variables	Categories	Frequency	Percentage
Age of women (years)	15–24	471	31.7
	25–34	724	50.3
	36–49	265	18.0
Educational attainment of women	No education	650	44.5
	Primary education	609	41.6
	Secondary education or above	202	13.9
Marital status of the women	Married	1,379	94.4
	Not married	82	5.6
Household-level wealth index	Poorest	294	20.2
	Poorer	309	21.1
	Middle	275	18.9
	Richer	257	17.4
	Richest	325	22.5
Religion	Orthodox	538	36.9
	Muslim	474	32.5
	Protestant	412	28.2
	Others*	36	2.4
Sex of child	Male	764	51.9
	Female	697	48.1
Age of child	6–11 months	476	32.1
	12–23 months	985	67.9
Number of under-five children in the household	One	634	42.9
	Two	680	46.6
	Three and above	147	10.5
Current breastfeeding status	No	215	15.6
	Yes	1,246	84.4
Mother’s ANC visit status	No	350	24.5
	At least one	1,110	75.5
Residence	Urban	413	28.4
	Rural	1,048	71.6
Regions**	Metropolitan	61	4.2
	Large centrals	1,267	86.6
	Pastoralist predominant	132	9.2

*Religions such as Catholic, traditional, and other religions. ANC: Antenatal care visit

**Regions: Metropolitan (Addis Ababa, Harari, and Dire Dawa), large central (Tigray, Amhara, Oromia, SNNPR), and predominately pastoralist regions (Afar, Benishangul-Gumuz, Gambelia, and Somali).

### Animal sources of iron-rich food consumption among 6–23 months old children in Ethiopia

**Fig 1:**
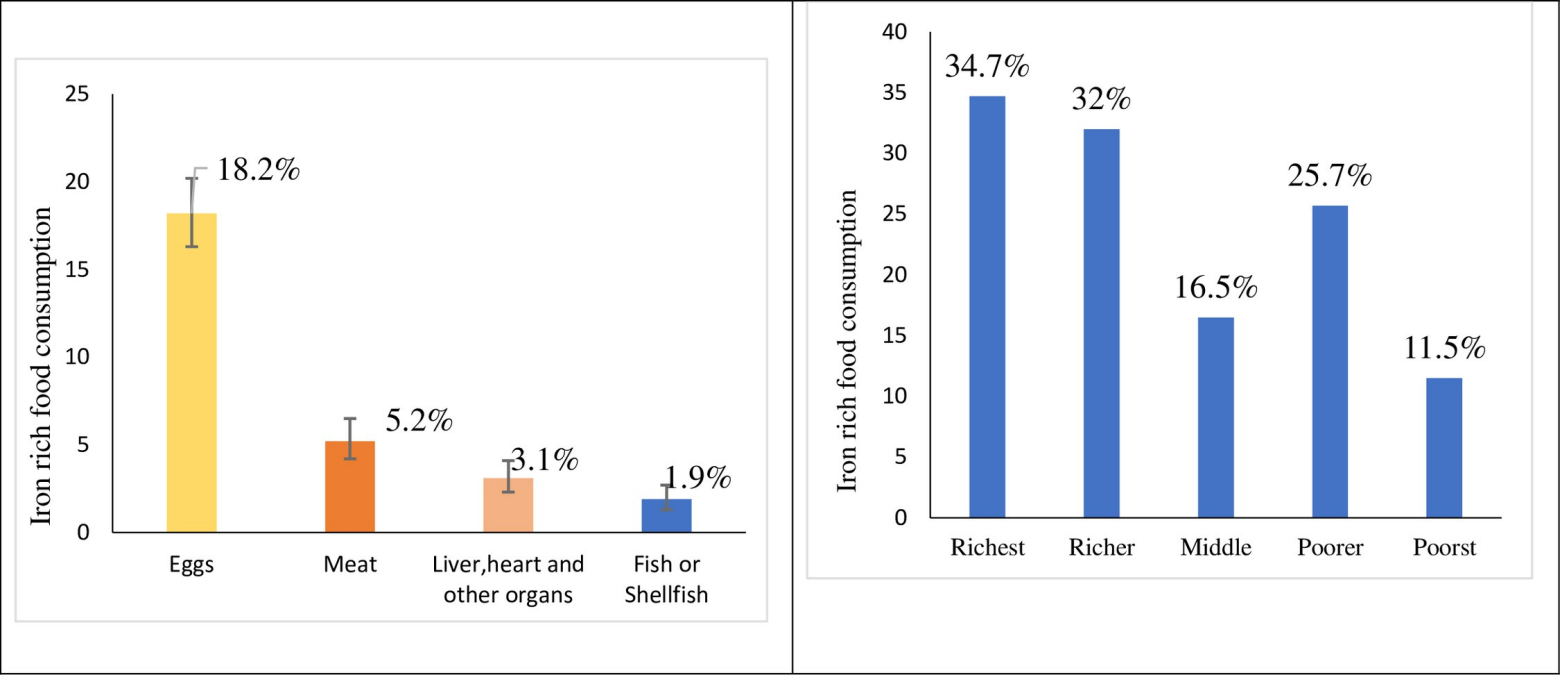
The bar graph shows the magnitude of iron-rich food consumption across types of food items and wealth status among children under the age of two years in Ethiopia.

About 24.2% (95% CI: 22.1, 26.5) of children 6–23 months were reported to consume iron-rich animal source foods the preceding day in our sample. This ranges from 0.3% in Dire Dawa to 37.8% in the Oromia region. From the four food item components, nearly one-fifth (17.7%) of children consumed eggs, however, only <2% of children consumed fish or shellfish in Ethiopia. More than one-third (34.7%) of children from the richest households consumed at least one iron-rich food while it was only 11.5% of children from the poorest households (Fig 1).

### Wealth-related inequalities of iron-rich animal source foods consumption among children under age two years in Ethiopia

In our study, there was a pro-rich wealth distribution of iron-rich animal source foods consumption among children in Ethiopia with a concentration index value of C = 0.25 (95% CI: 0.12, 0.37). This means that the higher socioeconomic groups have higher animal sources of iron-rich food consumption.

**Fig 2:**
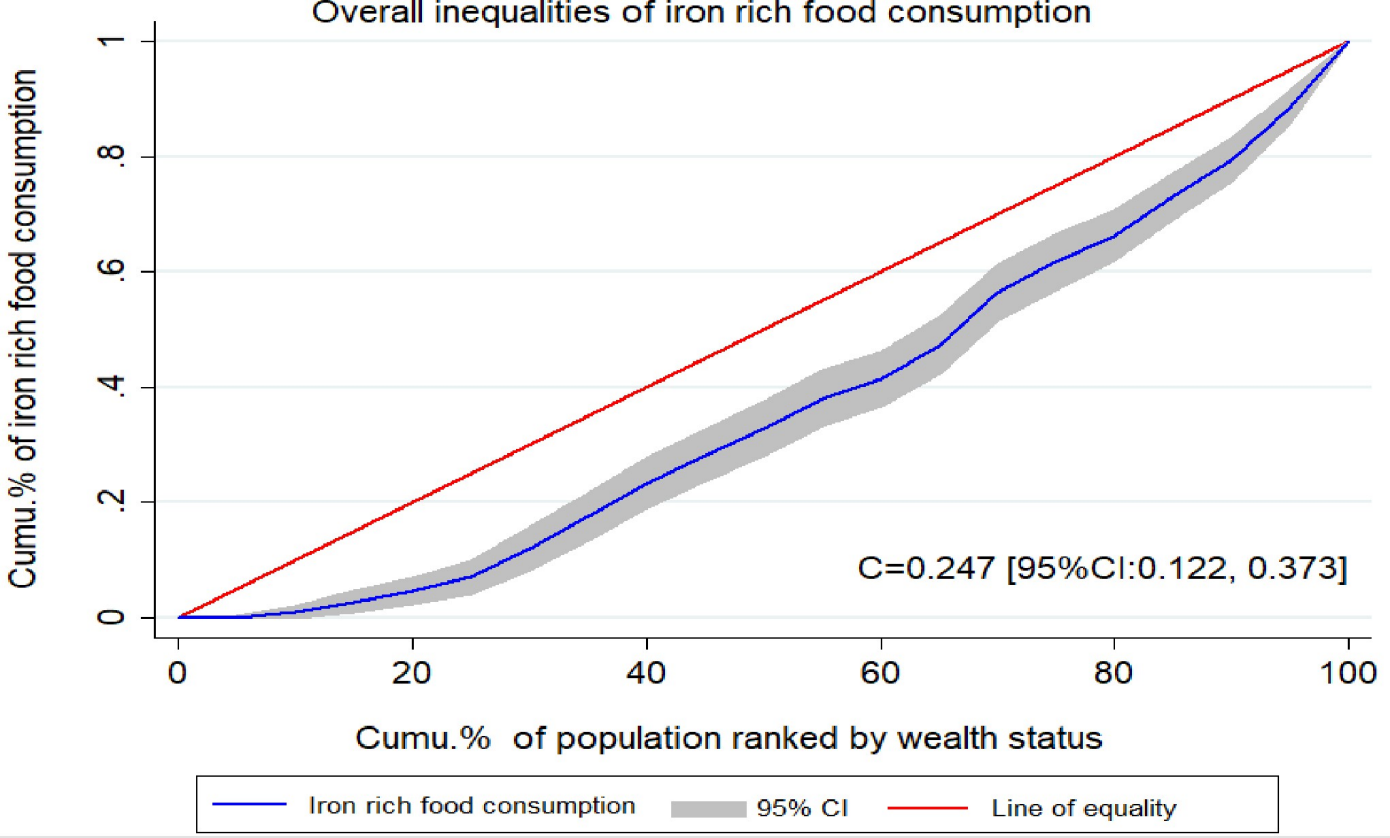
Overall wealth-related inequalities of iron-rich food consumption among children under age two years in Ethiopia.

The finding from the indices agrees with the results of the concentration curves. The concentration curve in Fig 2 also showed that the concentration graph of iron-rich food consumption was below the line of equality which indicates that the distribution was more concentrated in rich households (Fig 2).

### Factors that contribute to the wealth-related inequalities of iron-rich food consumption

In this study, about 70.04% of the wealth-related inequalities of iron-rich food consumption among children were explained by the combination of variables fitted in the Wagstaff model.

Children with mothers having primary or above education contribute 21% of the wealth-related inequality in the consumption of iron-rich animal sources of food among children. The elasticity result of Table 2 showed that a 1% increase in the proportion of women with education at primary levels or above, could increase wealth-related inequality of iron-rich food consumption among children by 21.5%, and 9% respectively.

**Table 2:** Decomposition of the concentration index of wealth-related inequalities for iron-rich animal source foods consumption among children aged 6–23 months in Ethiopia evidence by 2019 EMDHS.

Variables	Categories	Coefficient (95% CI)	Elasticity	C	Cont.^a^	%Cont.^b^
Age of women	15–24 (Ref.)					
	25–34	0.012 (-0.010, 0.032)	0.015	0.016	<0.001	<0.001
	35–49	0.002(-0.019, 0.023)	0.001	0.006	<0.001	<0.001
	Subtotal				<0.001	<0.001
Educational attainment of women	No education (Ref.)					
	Primary education	0.102(0.047, 0.156) **	0.215	0.114	0.024	9.72
	Secondary & above	0.188(0.103,0.272) ***	0.090	0.307	0.027	10.93
	Subtotal				0.052	21.05
Marital status	Not married (Ref.)					
	Married	0.005(-0.078, 0.084)	-0.192	0.034	-0.007	-2.83
Religion	^c^Others (Ref.)					
	Orthodox	0.008(-0.048, 0.065)	-0.012	0.174	-0.002	-0.81
	Muslim	-0.041(-0.065,0.056)	0.092	-0.188	-0.018	-7.29
	Subtotal				-0.020	-8.10
Age in months	6–11 months (Ref.)					
	12–23 months	0.071(0.028,0.113) *	0.201	0.027	0.006	2.43
Current breastfeeding status	No (Ref.)					
	Yes	0.011(-0.044,0.067)	0.117	0.056	0.007	2.83
ANC	No (Ref.)					
	Yes	0.056(0.003,0.110) *	0.114	0.316	0.036	14.57
No. of under-five children in the family	One (Ref.)					
	Two	0.004(-0.041, 0.049)	0.057	-0.118	-0.007	-2.83
	Three above	-0.049(-0.117,0.019)	-0.027	-0.293	0.008	-3.24
	Subtotal				0.001	0.40
Household-level wealth index	Poorest (Ref.)	0.091(-0.005, 0.176)	0.087	-0.488	-0.042	-17.00
	Middle	0.061 (-0.023, 0.144)	0.004	0.018	<0.001	<0.001
	Richer	0.141 (0.047, 0.234) **	0.091	0.260	0.024	9.72
	Richest	0.094 (0.012, 0.199) *	0.087	0.490	0.043	17.41
	Subtotal				0.025	10.12
Residence	Urban (Ref.)					
	Rural	-0.037(-0.111, 0.036)	-0.105	-0.406	0.043	17.41
Region	Pastoral predominant (Ref.)					
	Metropolis	0.067(0.007, 0.170) *	0.036	0.321	0.012	4.86
	Large central	0.066(0.009, 0.123) *	0.215	0.085	0.018	7.29
	Subtotal				0.030	12.15
	Total explained inequality				0.173	70.04
	Residual				0.074	29.96
	Overall inequality				0.247	100.00
	95% conf. interval			C = 0.247 (0.122, 0.373)		

* = P value < 0.05

** = P value < 0.01

*** = P value < 0.001

Ref. = Reference category; C = concentration index, ANC = Ante Natal Care

^a^ Cont.C (Contribution to concentration index) = C *Elasticity

^b^ %Cont = Percentage contribution to concentration index = (Cont.C/ Overall conc. index)*100

^c^Religions such as protestant, catholic, traditional, and other religions

Moreover, 12% of wealth-related inequality in the consumption of iron-rich animal sources of food was explained by living in the large central and metropolitan regions of Ethiopia. A 1% change from predominantly pastoral to a large central region could increase the wealth-related inequality of iron-rich animal sources of food consumption by 21.5%. Likewise, a similar percentage change in the urbanization of the pastoral population could contribute to wealth-related inequality by 3.6%.

Mothers who had at least one ANC visit during pregnancy explained 14.6% of the wealth-related inequalities in iron-rich food consumption among children. Additionally, the household wealth index could explain 10% of the inequalities of iron-rich animal sources of food consumption among children. Being older children (12–23 months child) explained 2.4% of the estimated pro-rich inequalities in iron-rich food consumption, with a concentration in wealthier groups (Table 2).

### Spatial analysis of iron-rich animal source foods consumption among children under age two years in Ethiopia

**Fig 3:**
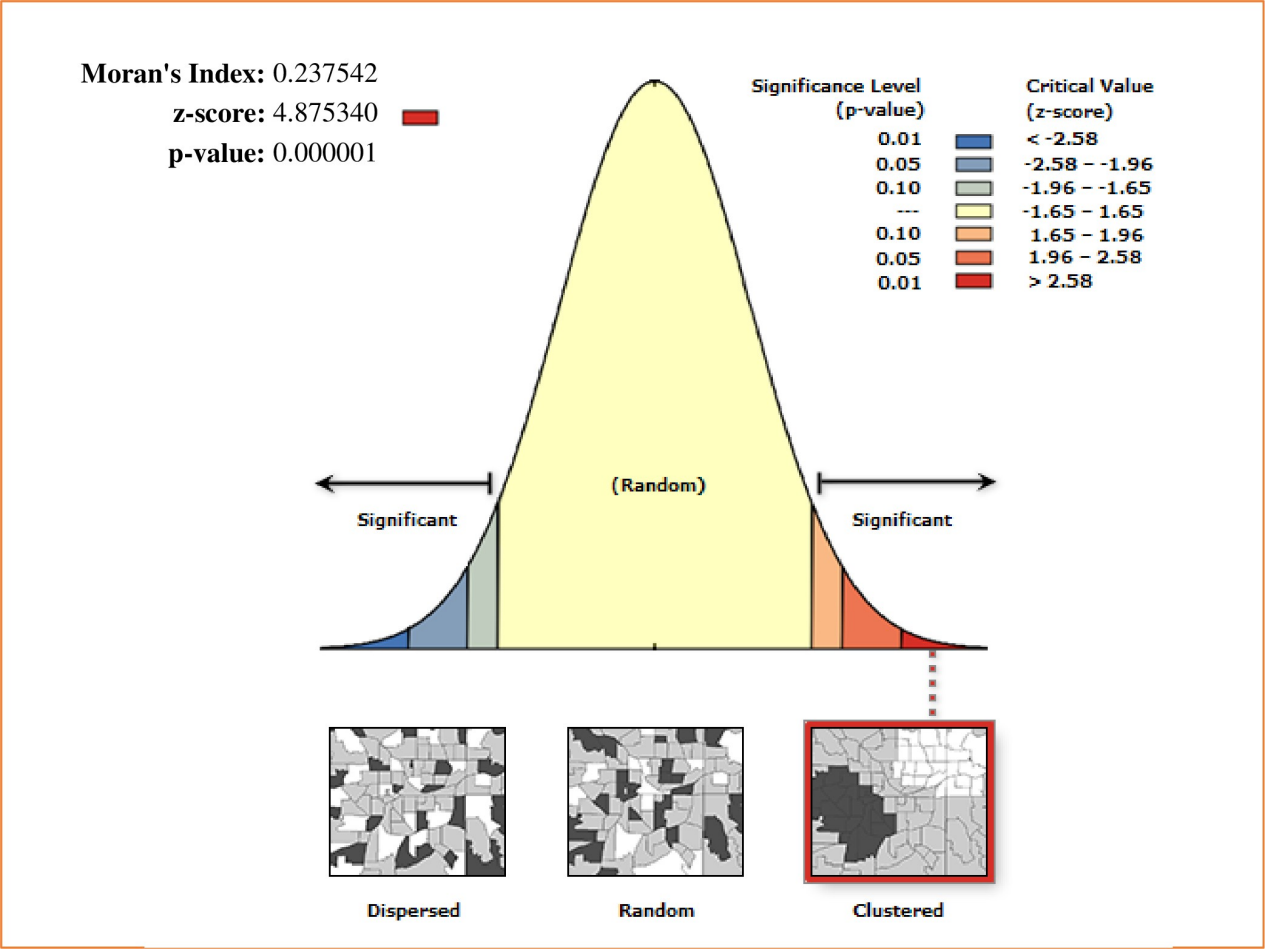
Spatial autocorrelation of iron-rich animal source foods consumption among children under age two years in Ethiopia. The base map for the shapefile was sourced from: https://gadm.org/download_country.html#google_vignette.

**Fig 4:**
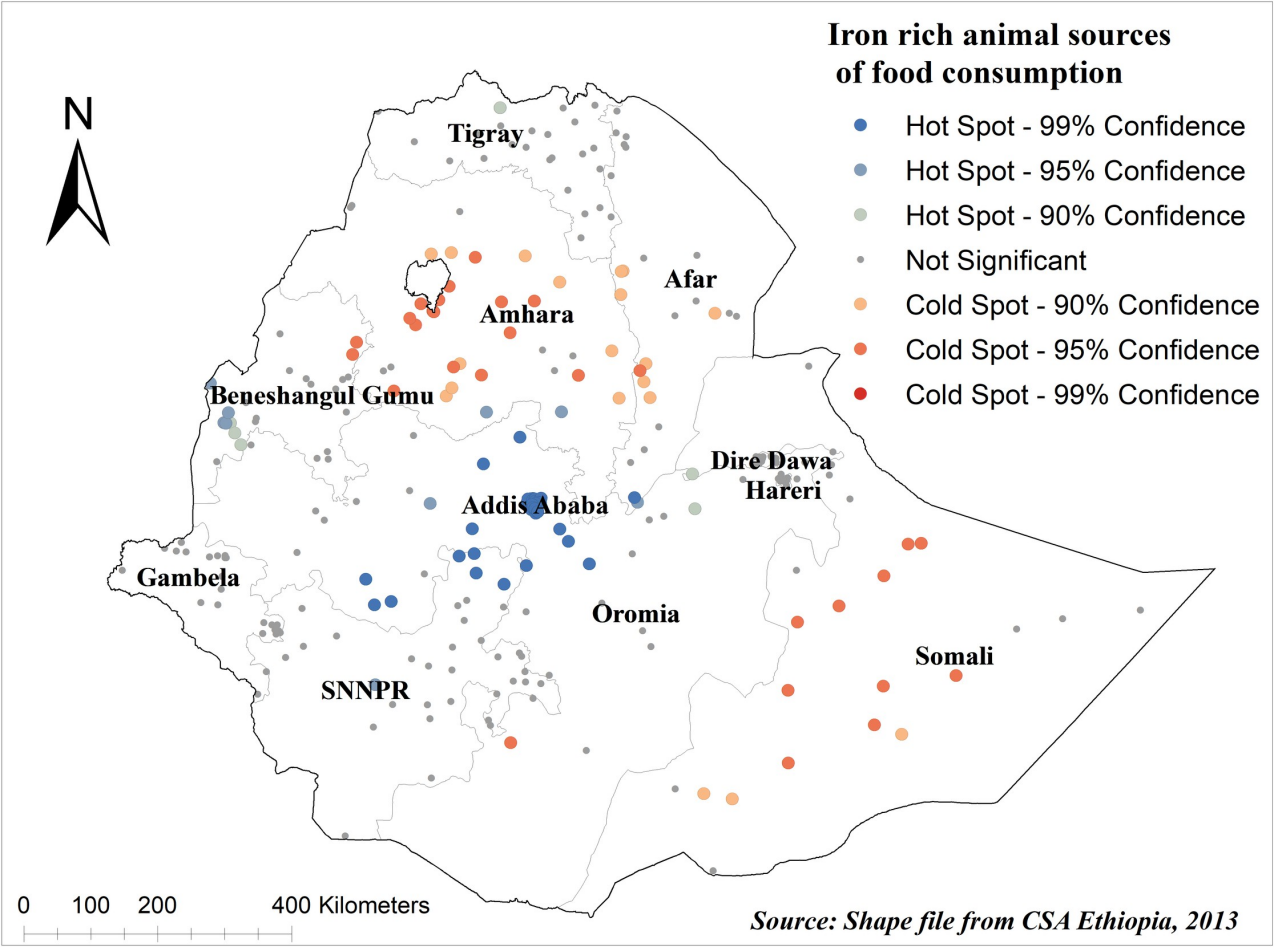
Hotspot analysis of iron-rich animal source foods consumption among children under age two years in Ethiopia. The base map for the shapefile was sourced from: https://gadm.org/download_country.html#google_vignette.

In this study, there was a significant spatial variation over regions of Ethiopia in iron-rich animal source foods consumption among children under age two years and was found to be clustered with Global Moran’s Index value of 0.238 (p<0.001) (Fig 3). Addis Ababa, Dire Dawa, Harare, Benishangul Gumuz, SNNPR, and Eastern Tigray regions are the hot spot areas, whereas most of Amhara, Eastern Afar, and Somali regions are the cold spot areas (Fig 4).

**Fig 5:**
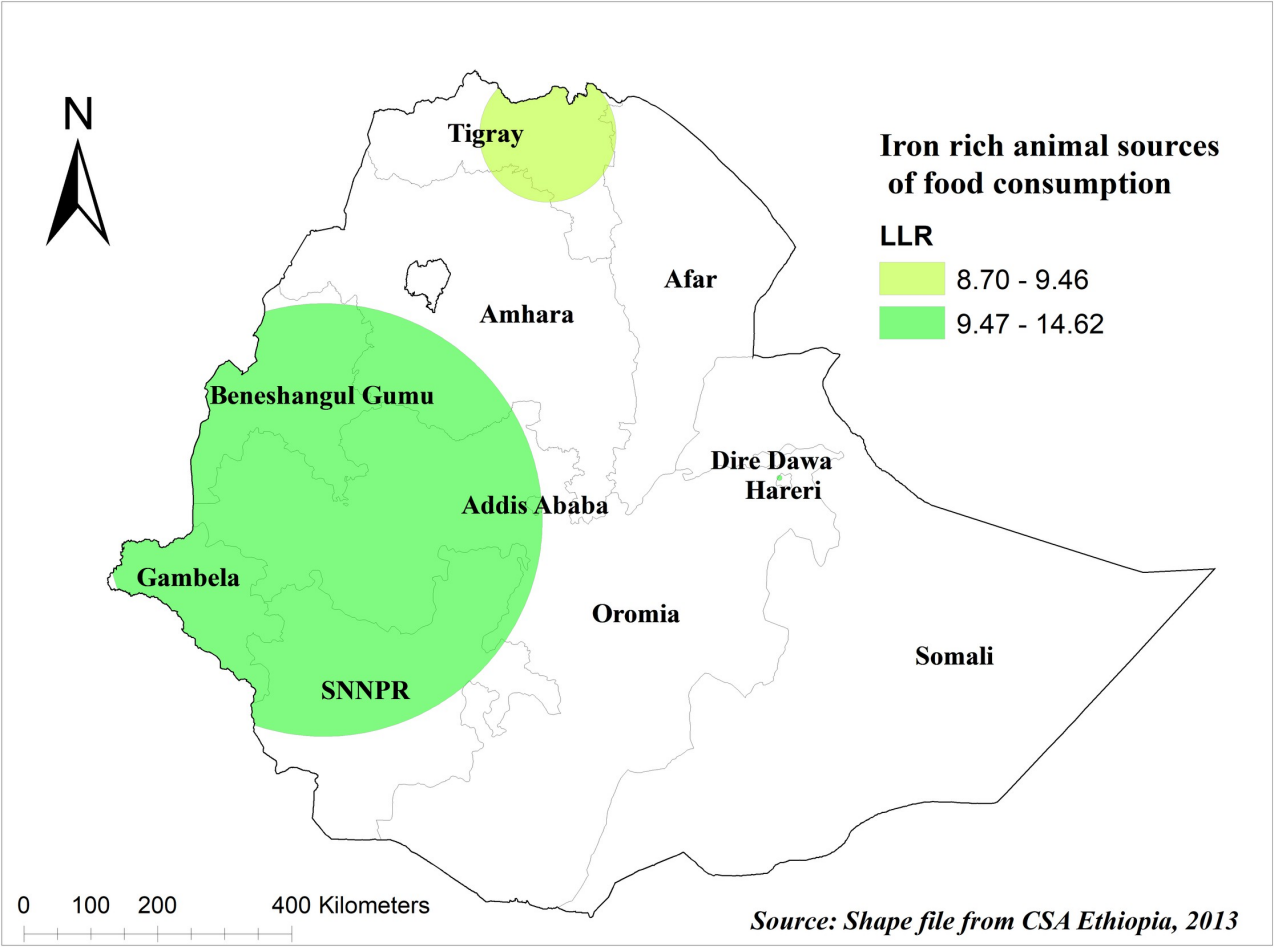
SaTscan analysis of iron-rich animal source foods consumption among children under age two years in Ethiopia, LLR: Log likely hood ratio. The base map for the shapefile was sourced from: https://gadm.org/download_country.html#google_vignette.

The SaTscan analysis of iron-rich animal source foods consumption among children in Ethiopia showed that 120 primary and 29 secondary clusters were detected for having a significant spatial window in iron-rich food consumption. These 120 primary clusters were centered at 8.759844 N, and 35.923489 E with a 324.89 km radius and located in Addis Ababa, Western Oromia, Gambelia, Benishangul Gumuz, and SNNPR regions (Fig 5).

Children who were found in these primary windows were 1.7 times more likely to consume iron-rich food than out of the window regions (RR = 1.7, P-value<0.001). whereas the secondary clusters are found in the smallest window and entirely in the Tigray region with a short radius of 13.985245 N, 38.954807 E, and 101.70 km radius (S1 Table and Fig 5).

**Fig 6:**
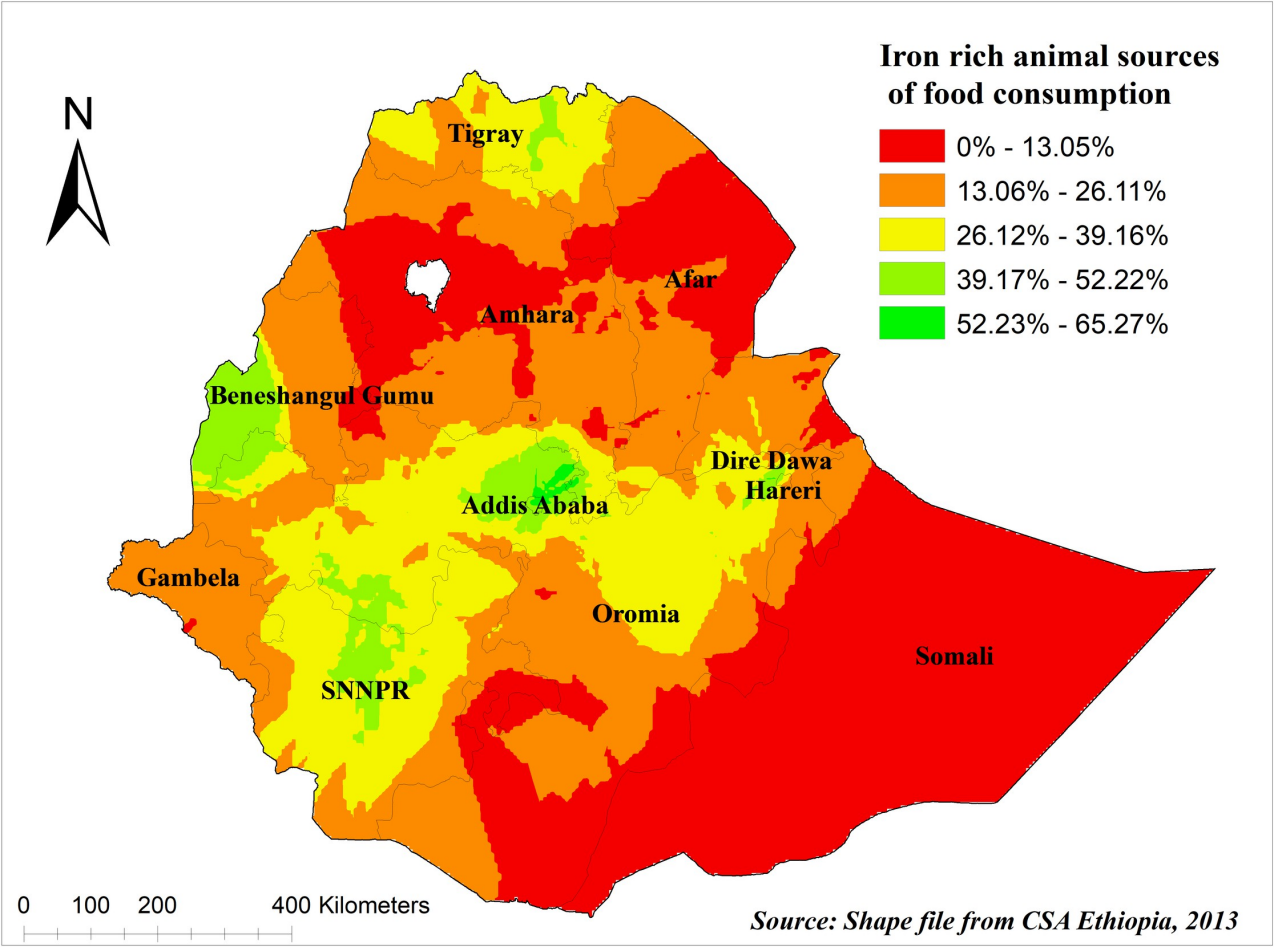
Kriging interpolation of iron-rich animal source foods consumption among children under age two years in Ethiopia. The base map for the shapefile was sourced from: https://gadm.org/download_country.html#google_vignette.

The predicted iron-rich animal source foods consumption among children in Ethiopia using Kriging interpolation methods showed that high-risk areas of predicted iron-rich food consumption range from 52.23% to 65.27% and are in Addis Ababa, Dire Dawa, and Harare regions. Whereas the lower predicted area was seen in Afar, Somali, and Northern and Western Amhara regions ranging from 0% to 13.05% (Fig 6).

## Discussions

The study aimed to assess the magnitude and the patterns of spatial and socio-economic inequalities in animal sources of iron-rich foods consumed by children aged 6–23 months in Ethiopia. We found that 24.2% of children consumed animal-source food items that are known to be iron-rich. Consumption was concentrated in wealthier groups (C = 0.25). This wealth-related inequality of iron-rich animal source foods consumption among children was attributable to maternal educational status, household wealth index, age of the child, having an ANC visit, and geographic regions children live in.

Our finding on the magnitude of animal sources of iron-rich food consumption was comparable with a report in Ethiopia (21.41%) [4] and Rwanda (23.56%) [44] that used similar food items to assess iron-rich food consumption. However, it is lower than the reports from several studies including reports from other East African countries such as Zimbabwe in 2016 (46%), Uganda in 2016 (40%), Malawi in 2015 (38%), and Kenya in 2014 (33%) [45–48], East Asia and the Pacific (62.5%) [49], and Bangladesh (48%) [50]. This might be due to differences in nutritional programs implemented in these countries [15]. For instance, Kenya is one of a few countries which are on track for the World Health Assembly undernutrition targets [51, 52] by implementing a health platform which integrated WASH (Water, Sanitation, and Hygiene) into complementary feeding sessions [52]. The socioeconomic and food culture differences between countries might have also played a role in these differences. The countries with a higher prevalence of iron-rich food consumption were found to have better access to iron-rich foods such as fish products and meat [53], which is partly attributed to their access to the sea. Despite Ethiopia having one of the largest livestock production [54] and having a high potential for fishery [55] in Africa, its consumption rate is very low [54, 55] accounting for less than 1% of total weight consumed [56]. It is mainly used for market purposes and household income generation rather than their home consumption by the family members [56, 57]. Moreover, a qualitative study in Ethiopia showed animal source foods are consumed in Ethiopia during special societal occasions since they are relatively costly and are not often considered an essential part of children’s daily diet [56]. A study in price patterns of animal sources of food in Ethiopia from 2007 to 2016 also showed that as a result of the increase the prices of the animal sources of food by 32% to 36% for three major food items eggs, milk, and meat, the consumptions were decreased by approximately 25% [57].

In most low-middle-income countries, the analysis of the actual nutrient densities of complementary foods has led to the identification of several issues in the content of micronutrients, including iron [14, 58]. The Ethiopian Federal Ministry of Health advises using local recipes for preparing complementary foods for children aged 6–23 months [59]. These traditional Ethiopian foods, such as *gruel*, *porridge*, *bulla*, *fetfet*, *kitta*, *and dabbo*, are made from grains such as teff, sorghum, barley, maize, wheat, enset, and legumes like broad beans, chickpeas, field peas, and lentils [59]. Furthermore, cereals and legumes in Ethiopia are utilized in the production of locally commercially available complementary foods. These products also include a vegetable protein concentrate and dried skimmed milk [60, 61]. However, it is recognised that the iron content of local foods and their bioavailability is limited as they are primarily plant-based [59]. A study conducted in Ethiopia for the development of local food-based complementary feeding recommends that maize, teff, and sorghum serve as the main sources of iron in the diets of breastfed children, functioning as complementary food items [14].

Moreover, in our study, there is also a pro-rich distribution reflecting the disproportionate concentration of iron-rich food in rich households. This is consistent with decomposition analysis studies conducted on micronutrients and child nutrition in India [15, 62], and Ethiopia [63].

Our finding of higher iron-rich animal sources of food consumption inequalities in educated mothers is supported by a study in India [15] and Afghanistan [6]. The Global Nutrition Report 2020 also pointed out that, the education gap contributes 7.7% of child nutrition inequalities [64]. This might be because educated mothers had greater health literacy [65], and the opportunity to participate in employment schemes that provided the capability to purchase nutrition-rich food items for their children and implement nutrition-diversified feeding habits [66].

In this study, the wealth inequality in iron-rich animal sources of food consumption was more pronounced among 12–23 months children as compared to 6–11 months children. This is in line with the previous studies in Ethiopia [4, 67]. The possible justification might be families of children in the early period would not commence complementary foods including animal sources of iron-rich foods for their children due to behavioural and cultural practices of the families [4, 67]. Mothers may perceive that the younger the children, the poorer the ability of the intestine to digest certain foods like meat [67]. Hence, they might not give age-appropriate complementary food to their young children (6–12 months). This is particularly important as commonly introduced complementary food items include grains (48%), fruits and vegetables (20%), food made from roots and tubers (19%), foods made from legumes and nuts (19%), cheese, yoghurt, and other milk products (14.6%), meat, fish, and poultry (6.5%) and eggs (14.3%) based on 2019 EMDHS [30]. Of these items, while meat, fish, poultry, and eggs are animal sources of food that provide heme iron, milk and milk products have limited iron [14, 22]. Since infants grow so quickly, iron needs are high in the first few months after birth to form red blood cells [7, 10]. Then, the late introduction of solid or semisolid foods into a baby’s diet is a common cause of iron deficiency in this age group [4]. Generally, wealthier families with children aged one year and above are better able to provide iron-rich animal sources of food for their children, unlike comparatively poorer families with children of the same age group. However, for children less than one year old, as they are more dependent on breastfeeding, the difference in family wealth status has no impact on the consumption of iron-rich animal sources of food.

In our study, there were no significant wealth-related inequalities in animal sources of iron-rich food intake between breastfed and non-breast feed children. While breast milk contains a small amount of iron, prolonged breastfeeding can lead to iron deficiency, particularly if breast milk replaces solid foods in the diet [12]. Therefore, supplementing bioavailable iron through complementary foods is recommended to prevent iron deficiency in breastfed infants during infancy [12]. In Ethiopia, breastfeeding until the age of 24 months is common with almost all children (96%) being breastfed at some point and 72% of children continuing breastfeeding until their second birthday [30]. Moreover, in our study, 85% of children were breastfed, but only one in four children (24%) aged 6–23 months consumed animal-source food items that are rich in iron. This should be improved, and meat products as well as iron-fortified foods should be introduced early to prevent iron deficiency and IDA in infants [7].

In our study, wealth inequalities in the consumption of iron-rich animal-source foods among children were evident among those whose mothers had received ANC visits. This is supported by a study in sub-Saharan Africa that shows mothers who have ANC visits during their pregnancy time have a higher chance of their children taking a minimum acceptable diet [67]. This is because having information and knowledge during follow up has a positive health behaviour like the utilization of iron-rich food for them and their child to prevent anaemia [44, 68].

In our study, 12% of the inequalities of iron-rich food consumption among children under the age of two years were explained by living in a metropolitan and large central region of Ethiopia. The spatial analysis also suggests that there is a significant spatial variation of iron-rich food consumption among regions in Ethiopia and clustered in Addis Ababa, Dire Dawa, SNNPR, and Eastern Tigray regions. This is in line with a study in Ethiopia [4]. A welfare monitoring survey conducted in Ethiopia also revealed that Dire Dawa, Addis Ababa, Harari, and Gambella were the regions where animal source food consumption was relatively high [69]. Meat was consumed by 54.3% of households in Tigray and 47.7% of households in Addis Ababa. Eggs were consumed by 38.8% of households in the capital, Addis Ababa, while the Somali region had the highest intake of milk, at 80.7% [69]. This geographical heterogeneity in animal sources of iron-rich food consumption across the regions of Ethiopia might be due to sociocultural differences such as pastoralist regions (Afar and Somali). For instance, a study in a pastoralist region in Ethiopia showed that the commonest food item usually consumed by the community was porridge which is made from wheat or corn flour and sometimes mixed with milk or butter which are not iron-rich food items [70]. Moreover, the difference in social norms and beliefs in child nutrition across the regions of Ethiopia have also their role [68]. The socioeconomic status which means availability, accessibility, and affordability of iron-rich foods across regions of Ethiopia due to the high cost of animal source foods, and low household income might be other determinants [4]. Although animal-source food consumption contributes not only to iron intake but also to other dietary qualities, decreased access and relatively higher price hampers its consumption in most low-income settings [69].

Moreover, there is an agroecological difference in nutrient intake from complementary foods consumed by young children [71]. A study on infant and young child feeding practices among children aged 6–23 months in rural Ethiopia revealed statistically significant differences in minimum dietary diversity intake among agroecological zones [72]. Specifically, 84% of children in midland agroecological zones achieved the WHO-recommended minimum dietary diversity, as compared to 72% of children residing in lowland agroecology zones [72]. Finally, it could be due to the high proportion of women in secondary and above education status in metropolitan (49.9%) and large central regions (13%) than in predominantly pastoral regions (6.3%) in our study.

### Limitations

Given that the data was obtained through cross-sectional self-reported interviews, there is a susceptibility to social desirability bias concerning the consumption of iron-rich animal food sources. To compute the wealth index of the households, the EMDHS used durable property which may not necessarily reflect the family’s income status that is applicable to purchase food items that are immediately required. Despite the data collection methods to measure iron-rich animal sources of food consumption employing standardised methods, the EMDHS data did not specify the amount of food consumed. In addition, although we used animal sources of food as an outcome variable for this study, there are plant-based food sources such as cereals, legumes, nuts, and seeds, for which information was not collected in DHS surveys. Therefore, it is worthwhile for future DHS surveys to consider collecting information on plant-based iron-rich food sources to allow examination of the full picture of iron-based dietary intake.

Despite the high bioavailability of heme iron, which is exclusively sourced from animal foods and is relatively free from inhibitors like phytate, and thus more readily absorbed by the body, the consumption of other dietary components, including those that promote or inhibit iron uptake, was not reported. Therefore, the results need to be interpreted with caution. In addition, some important determinants of iron-rich animal source food consumption such as birth weight and gestational age were not collected.

## Conclusion

The magnitude of iron-rich animal source foods consumption among children 6–23 months old in Ethiopia is low. The consumption was disproportionately concentrated in the rich households (pro-rich concentration). Inequalities in iron-rich animal source foods consumption were more apparent among the higher educational status of the mother, being 12–23 months of age, having ANC visits, and living in large central and metropolitan regions. Regions like Afar, Eastern parts of Amhara, and Somali regions should be considered priority areas for nutritional interventions for increasing iron-rich food consumption among children.

## Supporting information

S1 Table Significant spatial clusters of iron-rich animal source foods consumption among children under age two years in Ethiopia, 2019 EMDHS. (DOCX)
