# Supplementary material for: Socio-economic and spatial inequalities in animal sources of iron-rich foods consumption among children 6–23 months old in Ethiopia: A decomposition analysis
Source: PLOS Glob Public Health. 2024 May 16;4(5):e0003217. doi: 10.1371/journal.pgph.0003217 (PMC11098381; doi:10.1371/journal.pgph.0003217)
Supplement: S1 Table — (DOCX) [file pgph.0003217.s001.docx]

S1 Table. Significant spatial clusters of iron-rich animal source foods consumption among under two years of age children in Ethiopia, 2019 EMDHS.

| **Clusters** | **Enumeration areas**  **(clusters) detected** | **Coordinate/radius** | **Population** | **Cases** | **RR** | **LLR** | **P-value** |
| --- | --- | --- | --- | --- | --- | --- | --- |
| 1^ry^ (120) | 120, 92, 93, 94, 168, 169, 167, 97, 98, 194, 96, 208, 86, 207, 91, 209, 87, 164, 211, 155, 95, 221, 212, 195, 226, 225, 156, 230, 222, 227, 223, 170, 150, 119, 224, 213, 154, 201, 228, 166, 153, 214, 161, 152, 174, 147, 149, 229, 148, 158, 200, 157, 218, 163, 77, 112, 206, 217, 171, 160, 179, 176, 80, 220, 215, 177, 204, 79, 99, 162, 180, 196, 52, 146, 191, 189, 165, 72, 216, 205, 159, 73, 190, 173, 178, 203, 76, 259, 262, 260, 261, 175, 274, 257, 219, 70, 258, 275, 276, 263, 277, 265, 256, 264, 270, 279, 267, 278, 266, 273, 100, 198, 280, 192, 71, 271, 268, 53, 272, 269 | (8.759844 N, 35.923489 E) / 324.89 km | 555 | 161 | 1.70 | 14.62 | <0.001 |
| 2^nd^ (29) | 232, 231, 246, 237, 240, 238, 235, 253, 243, 242, 233, 239, 234, 11, 12, 14, 13, 2, 16, 10, 7, 17, 1, 3, 6, 15, 23, 25, 9 | (13.985245 N, 38.954807 E) / 101.70 km | 86 | 35 | 2.28 | 9.46 | <0.001 |
